# Re-evaluation of penicillin and ceftriaxone MIC results to predict susceptibility to the oral cephalosporin, cefpodoxime, in *Streptococcus pneumoniae* clinical isolates from the United States according to CLSI guidelines (2019–2021)

**DOI:** 10.1128/jcm.00027-25

**Published:** 2025-06-24

**Authors:** Rodrigo E. Mendes, Jessica V. Pierce, Kelly Wright, Michael D. Huband, Mariana Castanheira

**Affiliations:** 1 Element Iowa City (JMI Laboratories) 138461, North Liberty, Iowa, USA; 2 Paratek Pharmaceuticals, King of Prussia, Pennsylvania, USA; Children's Hospital Los Angeles, Los Angeles, California, USA

**Keywords:** respiratory infections, CABP, surrogate, omadacycline

## Abstract

Clinical and Laboratory Standards Institute (CLSI) M100Ed24 (2024) states that *Streptococcus pneumoniae* susceptible to oral penicillin can be considered susceptible to various β-lactams, including oral cephalosporins. However, surrogacy guidance is not available for isolates nonsusceptible to oral penicillin. Instead, such isolates require specific MIC testing and interpretations for reporting susceptibility to oral cephalosporins, for which susceptibility testing is limited in most automated systems, and consequently restricts information pertaining to oral cephalosporins at an individual institution. This study evaluated the ability of the penicillin breakpoints to predict cefpodoxime and ceftriaxone susceptibilities against a recent collection of *S. pneumoniae* from United States hospitals according to CLSI guidelines. The susceptible breakpoint for oral penicillin (≤0.06 mg/L) predicted susceptibility to cefpodoxime and ceftriaxone. However, when isolates were nonsusceptible to oral penicillin (MIC, >0.06 mg/L), susceptibility to cefpodoxime could not be predicted due to a low categorical agreement (CA) (78.4%). Parenteral penicillin breakpoints also could not predict cefpodoxime susceptibility due to the elevated number of very major errors and a CA of 76.8%; however, these breakpoints could still be used as a surrogate marker to predict ceftriaxone susceptibility. Finally, ceftriaxone could be used for surrogate testing of cefpodoxime by applying breakpoints (≤0.25 mg/L for susceptible; 0.5 mg/L for intermediate; ≥1 mg/L for resistant) lower than the current clinical cutoffs. These analyses showed that isolates nonsusceptible to oral penicillin cannot be considered susceptible to cefpodoxime, and caution should be used when prescribing oral cephalosporins for the empiric treatment of community-acquired bacterial pneumonia.

**IMPORTANCE:**

Susceptibility results to oral cephalosporins are rarely available to guide therapy due to the limited number of drugs evaluated on common automated antimicrobial susceptibility testing (AST) systems (i.e., Vitek 2, MicroScan, and Phoenix). In addition, disk diffusion methods for determining *Streptococcus pneumoniae* susceptibility to β-lactam agents are not reliable, and a quantitative method, such as broth microdilution or gradient strips, is required. In addition, the epidemiology and serotypes of *S. pneumoniae* are constantly evolving; therefore, this work provides a re-evaluation of surrogacy testing for β-lactam agents against *S. pneumoniae* recently recovered from United States laboratories. The data provide the possible use of ceftriaxone MIC for determining cefpodoxime susceptibility. This should be of interest to microbiology laboratories and the scientific community.

## INTRODUCTION

According to the World Health Organization, lower respiratory infections were the sixth most common cause of death in high-income countries in 2019 and among the top 5 causes of death in low-, lower-middle, and upper-middle income groups (1). More specifically, pneumonia is a leading infectious cause of hospitalization and death among adult patients in the United States (2), and *Streptococcus pneumoniae* remains the most common cause of community-acquired bacterial pneumonia (CABP) (2, 3). Although there is a trend toward the reduction of mortality due to pneumonia over time, the attributable mortality of pneumococcal CABP has not changed in the last two decades, in conjunction with an increase in the rate of hospitalization and more severe forms of CABP (4).

CABP is often treated empirically, and the Infectious Diseases Society of America (IDSA) recommends β-lactam, macrolide, doxycycline, or respiratory quinolone monotherapy for outpatients with non-severe infection, whereas combination therapy is recommended for patients with comorbidities (5). In addition, when intravenous therapies are utilized, various treatment guidelines and stewardship programs now recommend switching to oral antibiotics when patients demonstrate clinical improvement, have adequate oral intake and gastrointestinal absorption, and are hemodynamically stable (5–7). In the United States, the most common oral β-lactams used in this setting are amoxicillin, amoxicillin-clavulanate, cefdinir, cefuroxime, and cefpodoxime (8, 9). However, even when *S. pneumoniae* is recovered from clinical specimens and available for antimicrobial susceptibility testing (AST), susceptibility results to recommended oral cephalosporins are rarely available to guide therapy due to the limited number of drugs evaluated on common automated AST systems (i.e., Vitek 2, MicroScan, and Phoenix). In addition, disk diffusion methods for determining *S. pneumoniae* susceptibility to β-lactam agents are not reliable, and quantitative (MIC) methods, such as gradient strip diffusion or broth microdilution, are required (10).

The Clinical and Laboratory Standards Institute (CLSI) M100Ed34 document states that susceptibility to oral penicillin (MIC, ≤0.06 mg/L) can be used as a surrogate test for the determination of *S. pneumoniae* susceptibility to several oral and parenteral β-lactam agents, including oral cephalosporins (10). However, the epidemiology and serotypes of *S. pneumoniae* causing CABP and invasive pneumococcal disease are constantly evolving, and therefore, the use of surrogate testing should be re-evaluated using contemporary isolates (11). In addition, a recent study reported that 16.1% to 48.7% of clinical *S. pneumoniae* causing CABP through the nine United States census regions were nonsusceptible to oral penicillin (MIC, ≥0.12 mg/L) (12). In cases such as these, *S. pneumoniae* susceptibility to oral and parenteral β-lactam agents cannot be predicted for penicillin-nonsusceptible isolates (MIC, ≥0.12 mg/L), and there is no CLSI guidance for alternative surrogate testing. Here, we re-evaluated the use of penicillin MIC results and breakpoints to predict the susceptibility of oral cephalosporins (i.e., cefpodoxime) using a contemporary, multicenter and nationwide collection of *S. pneumoniae* recovered from patients seen in United States centers (2019–2021) according to CLSI guidelines. Ceftriaxone MIC results and breakpoints were also evaluated to understand if these could be used as a surrogate for determining susceptibility to oral cephalosporins.

## MATERIALS AND METHODS

A total of 1,035 *S*. *pneumoniae* isolates from 31 medical centers located in the nine United States census regions collected between 2019 and 2021 as part of the SENTRY Antimicrobial Surveillance Program were tested by CLSI broth microdilution methods and interpreted according to current CLSI guidelines, M100Ed34 (2024) (10). Broth microdilution MIC testing of penicillin, cefpodoxime, and ceftriaxone was conducted using the Clinical and Laboratory Standards Institute (CLSI) M07Ed13 (2024) methodology. Cation-adjusted Mueller-Hinton broth (CAMHB) media lots were obtained each year from Becton-Dickinson (BBL; Franklin Lakes, New Jersey). Freshly prepared CAMHB was used for susceptibility testing. Agents were obtained from commercial sources, including Patheon (Bend, Oregon), Sigma-Aldrich (St. Louis, Missouri), Toku-E (Bellingham, Washington), and the United States Pharmacopeia (Rockville, Maryland). Quality control (QC) results and breakpoint interpretations utilized CLSI M100ED34 (2024).

Isolates were from patients with CABP (*n* = 933), bloodstream infections (*n* = 72), or nosocomial pneumonia (*n* = 33), and additional information related to this collection can be found as previously published (12). Data analysis for surrogacy was performed according to the comparison guidelines found in CLSI document M52Ed1 (2015) (13), and scattergrams were generated for penicillin versus cefpodoxime, penicillin versus ceftriaxone, and ceftriaxone versus cefpodoxime. Error rates and categorical agreements were calculated using current CLSI guidelines and breakpoints per M100Ed34 (2024) (Table 1) (10). Specifically, a very major error (false-susceptible) was defined as the number of susceptible results obtained by the surrogate, but resistant by the testing agent, and calculated based on this number divided by the number of resistant results by the surrogate agent; a major error was defined as the number of resistant results generated by the surrogate agent, but susceptible by the testing agent, and calculated based on this number, divided by the number of susceptible results obtained by the surrogate agent; a minor error was defined as intermediate results by the surrogate agent and susceptible or resistant by the testing agent or vice versa, and calculated based on this number divided by the total number of isolates tested (i.e., 1,035). Categorical agreement rate was calculated based on the number of results with the same categorical interpretations divided by the total number of results. Hypothetical breakpoints for ceftriaxone were attempted as a possible surrogate marker to predict cefpodoxime susceptibility based on the generation of lowest possible error rates. A categorical agreement rate of ≥90.0%, and very major, major, and minor error rates of ≤3%, ≤3%, and ≤10%, respectively, were considered acceptable (13, 14).

**TABLE 1:** CLSI M100Ed34 (2024) breakpoints for agents evaluated in this study

Agent	Category (MIC in mg/L)		
	Susceptible	Intermediate	Resistant
Oral penicillin	≤0.06	0.12–1	≥2
Parenteral penicillin (nonmeningitis)	≤2	4	≥8
Cefpodoxime	≤0.5	1	≥2
Ceftriaxone (nonmeningitis)	≤1	2	≥4

## RESULTS

**Fig 1:**
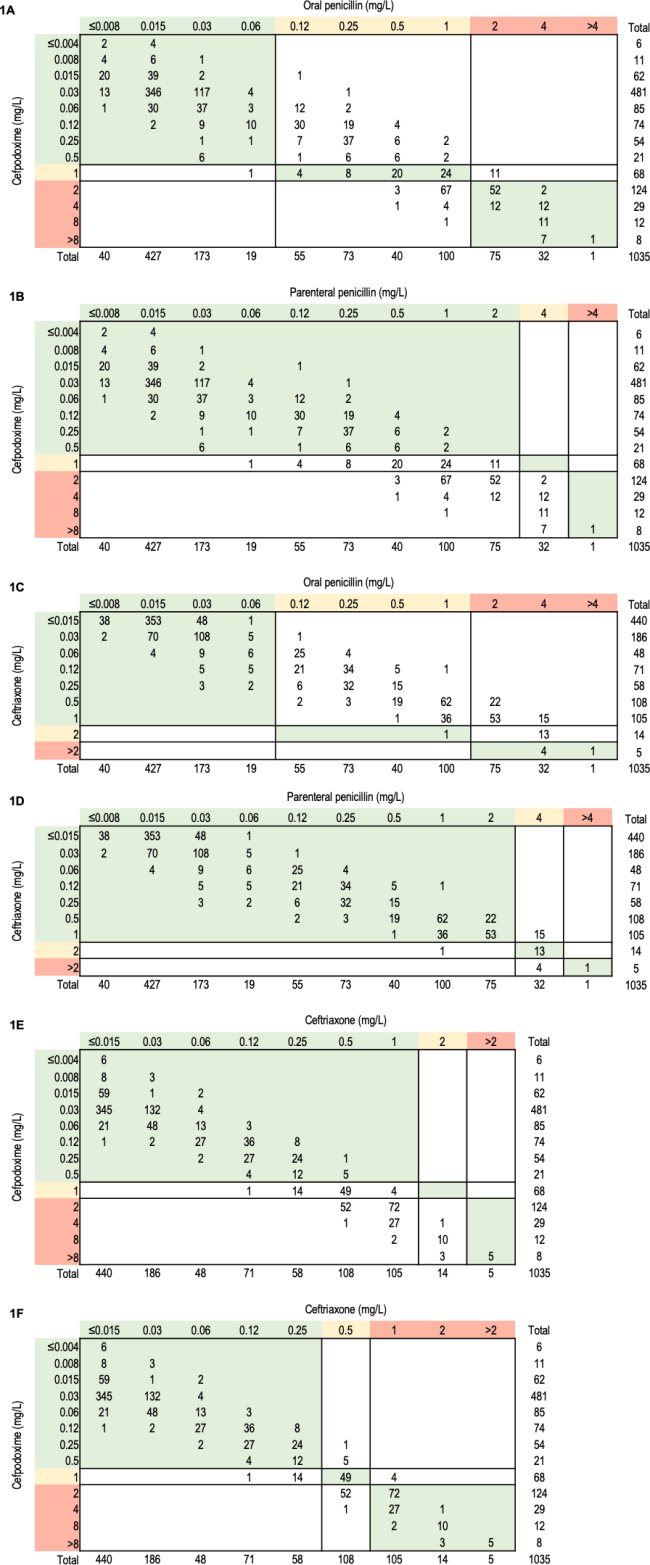
MIC values highlighted in green, yellow, and orange represent susceptible, intermediate and resistant categories, respectively. Cells containing the number of occurrences for every MIC correlation highlighted in green are considered categorical agreements. (A) Scattergram comparing the MIC results between oral penicillin and cefpodoxime. Cells were shaded to represent the susceptibility categorization based on the CLSI M100Ed34 (2024) breakpoints for oral penicillin and cefpodoxime. (B) Scattergram comparing the MIC results between parenteral penicillin and cefpodoxime. (C) Scattergram comparing the MIC results between oral penicillin and ceftriaxone (nonmeningitis). (D) Scattergram comparing the MIC results between parenteral penicillin and ceftriaxone (nonmenigitis). (E) Scattergram comparing the MIC results between ceftriaxone (nonmenigitis) and cefpodoxime. (F) Scattergram comparing the MIC results between ceftriaxone (nonmenigitis) and cefpodoxime using tentative ceftriaxone breakpoints to predict cefpodoxime susceptibility.

Table 2 shows a summary of error rates and categorical agreement results obtained when performing a comparison analysis between β-lactam agents tested against *S. pneumoniae* isolates. Scattergrams for surrogacy analysis performed can be observed in Fig. 1A through E. When using the CLSI M100Ed34 (2024) breakpoints for oral penicillin to predict susceptibility to cefpodoxime, 21.6% minor errors, no major or very major errors, and a categorical agreement of 78.4% were observed (Table 1; Fig. 1A). Using the CLSI breakpoints for parenteral penicillin to predict susceptibility to cefpodoxime generated 9.7% minor errors, no major errors, but elevated very major errors, and a categorical agreement of 76.8% (Table 2; Fig. 1B).

**TABLE 2:** Summary of categorical agreement and error rates between evaluated β-lactam agents

Surrogate versus testing agent^*a*^	% of error rate (N of errors/denominator)^*c*^			
	Very major	Major	Minor	% CA^*c*^
Oral penicillin versus cefpodoxime	0.0 (0)^*d*^	0.0 (0)	**21.6 (224)**	**78.4 (811)**
Parenteral penicillin versus cefpodoxime	**NC (140/1)** ^*c*^	0.0 (0)	9.7 (100)	**76.8 (795)**
Oral penicillin versus ceftriaxone	0.0 (0)^*d*^	**13.7 (90/659)**	**27.1 (280)**	**64.3 (665)**
Parenteral penicillin versus ceftriaxone	0.0 (0)^*d*^	0.0 (0)	1.9 (20)	98.1 (1015)
Ceftriaxone versus cefpodoxime	**NC (154/5)** ^*c*^	0.0 (0)	7.9 (82)	**77.2 (799)**
Ceftriaxone versus cefpodoxime^*b*^	0.0 (0)^*d*^	0.0 (0)	7.5 (78)	92.5 (957)

^*a*^ Evaluation of the susceptibility of each former agent (surrogate) to predict susceptibility of the latter. Values in bold were considered unacceptable, otherwise they were considered acceptable.

^*b*^ Error rates and categorical agreement when applying a tentative breakpoint for ceftriaxone (≤0.25 mg/L for susceptible; 0.5 mg/L for intermediate; ≥1 mg/L for resistant) to predict susceptibility to cefpodoxime.

^*c*^ Denominator described when applicable. The denominator for calculation of very major errors was considered as the total number of resistant results obtained by the surrogate agent; the number of susceptible results obtained by the surrogate agent was used as denominator for calculation of major errors. Calculation of minor and categorical agreement rates used the total number of results obtained (i.e., 1,035) as denominator. The very major errors (false-susceptibility) observed for cefpodoxime when using parenteral penicillin and ceftriaxone MIC results were not calculated (NC) due to the small number of resistant isolates when using their respective breakpoints.

^*d*^ The lack of very major errors for oral penicillin support recommendations to use susceptibility to oral penicillin as a one-way surrogate for cefpodoxime and ceftriaxone and similarly parenteral penicillin as a one-way surrogate for ceftriaxone.

The use of oral penicillin to predict susceptibility to ceftriaxone generated 27.1% minor errors, 13.7% major errors, no very major errors, and a categorical agreement of only 64.3% (Table 2; Fig. 1C). A categorical agreement of 98.1% and error rates of 0.0%–1.9% were obtained when using the parenteral penicillin breakpoint to predict ceftriaxone susceptibility.

The use of CLSI M100Ed34 (2024) non-meningitis breakpoints for ceftriaxone to predict susceptibility to cefpodoxime produced 7.9% and 0.0% of minor and major errors, but a significant number of very major errors, with a categorical agreement of 77.2% (Table 2; Fig. 1E). When applying hypothetical and lower breakpoints for ceftriaxone (i.e., ≤0.25 mg/L for susceptible, 0.5 mg/L for intermediate, and ≥1 mg/L for resistant) with the current cefpodoxime breakpoints to evaluate the possibility of using ceftriaxone MIC results to predict cefpodoxime susceptibility, a minor error rate of 7.5% was obtained, with no major or very major errors. In addition, a categorical agreement of 92.5% was observed (Table 1; Fig. 1F).

## DISCUSSION

The CLSI M100 guideline has stated for decades that *S. pneumoniae* susceptible to oral penicillin (MIC, ≤0.06 mg/L) can be considered susceptible to several oral and parenteral β-lactam agents, including oral cephalosporins (10). The epidemiology, serotype, serotype replacement, and antimicrobial susceptibility profiles of *S. pneumoniae* are constantly evolving, which warrants continuous surveillance of resistance and re-evaluation of previously established surrogacy rules used by clinical microbiology laboratories. The data presented here confirm that the oral penicillin breakpoints for *S. pneumoniae* still cannot provide a complete surrogate function for determining the susceptibility of cefpodoxime (minor error, 21.6%) and ceftriaxone (minor error, 27.1%; and major error, 13.7%) due to the elevated error rates. However, the one-way surrogacy recommendation (i.e., isolates susceptible to oral penicillin can be considered susceptible to other β-lactams) remains valid for the two agents evaluated here, cefpodoxime and ceftriaxone, given no occurrence of very major errors. These results are likely because isolates susceptible to oral penicillin should not possess β-lactam resistance mechanisms.

However, the use of one-way surrogacy with the susceptible breakpoint for oral penicillin to determine susceptibility to either cefpodoxime or ceftriaxone is limited due to the high number of isolates that are nonsusceptible to oral penicillin (36.3% in this study). In cases of nonsusceptibility to oral penicillin, the surrogacy approach is not viable, and susceptibility results for oral and parenteral β-lactams require individual quantitative MIC testing for each agent. Furthermore, the susceptible breakpoint for parenteral penicillin cannot be used to predict cefpodoxime susceptibility due to the number of very major errors (false-susceptible). In contrast, the parenteral penicillin breakpoints were predictive of ceftriaxone susceptibility with acceptable error rates (0%–1.9%) and elevated categorical agreement (98.1%). However, penicillin and ceftriaxone are usually available for AST in many automated systems, which is not the case for several oral cephalosporins, highlighting the need for improved surrogate testing in this case.

The current high number of *S. pneumoniae* nonsusceptible to oral penicillin minimizes the use of penicillin as surrogate testing, and these nonsusceptible isolates require specific AST for determining the susceptibility for oral and parenteral cephalosporins. This scenario challenges routine clinical microbiology laboratories due to the absence of oral cephalosporins in automated systems. Despite these AST challenges, clinical guidelines recommend amoxicillin or doxycycline as options for empiric treatment of CABP in outpatients without comorbidities, and amoxicillin-clavulanate or select oral cephalosporins in combination with a macrolide and doxycycline in patients with comorbidities. The rationale for these latest IDSA recommendations is that these combinations should effectively target macrolide- and doxycycline-resistant *S. pneumoniae*, as β-lactam resistance is less common (5). However, while *in vitro* resistance to amoxicillin-clavulanate and ceftriaxone is less common, 23.3% of isolates included in this study were nonsusceptible to cefpodoxime according to current CLSI M100Ed34 (2024) breakpoints. Moreover, a previous study reported cefpodoxime nonsusceptibilities of 46.7% and 38.1% when isolates were also nonsusceptible to macrolides or doxycycline, respectively (12). These data indicate the need for re-evaluation of oral cephalosporins for the empiric treatment of CABP in addition to potential guideline updates for surrogacy AST.

Ceftriaxone cutoffs (≤0.25 mg/L for susceptible; 0.5 mg/L for intermediate; ≥1 mg/L for resistant) lower than the current CLSI M100Ed34 (2024) breakpoints showed acceptable results when used to predict cefpodoxime susceptibility in *S. pneumoniae* with acceptable categorical agreement (92.5%) and low error rates (0.0%–7.5%). Similar findings were reported by Murphy et al. (15) when using the meningitis breakpoints for cefotaxime (≤0.5 mg/L for susceptible; 1 mg/L for intermediate; ≥2 mg/L for resistant) as a surrogate marker for cefdinir susceptibility in a single-center study (15). The correlation of susceptibility categorization between oral and intravenous cephalosporins when applying lower breakpoints for the latter is likely explained by distinct pharmacokinetic properties. For example, the maximum free drug concentration of intravenous ceftriaxone is sixfold higher than oral cefpodoxime. Similarly, ceftriaxone has a half-life twofold longer than cefpodoxime. Since both ceftriaxone and cefpodoxime have the same pharmacodynamic target (>40% time above MIC), ceftriaxone likely has a higher target attainment than cefpodoxime (16, 17).

It is important to emphasize that the MIC comparison analysis presented here assumes that the breakpoints published by CLSI M100Ed34 (2024) remain appropriate and can accurately differentiate between susceptible and resistant isolates. However, breakpoints for older agents, including those evaluated here, were established mostly based on MIC distributions, pooled clinical data, and limited pharmacokinetic/pharmacodynamic analysis (18). In addition, randomized clinical trial data evaluating the use of oral cephalosporins for treating CABP are limited, with some studies reporting favorable data related to early switch from parenteral to oral therapy published during the 1990s (19, 20). These trials were performed when pneumococcal nonsusceptibility to oral penicillin was approximately 50% lower than current resistance rates (23.6%(21) and 36.3%, respectively) and underscore the importance of considering more active agents for empiric therapy, such as omadacycline and lefamulin, which have a different mode of action (protein synthesis inhibitor) and potent *in vitro* activity against *S. pneumoniae* isolates (>99% susceptible when applying FDA interpretive criteria) (12).

This study confirms the use of the current susceptible breakpoint for oral penicillin (≤0.06 mg/L) as a one-way only surrogacy AST to predict susceptibility to cefpodoxime and ceftriaxone against a contemporary collection of *S. pneumoniae* clinical isolates from multiple centers in the US, as indicated by the CLSI M100Ed34 (2024) document. However, isolates nonsusceptible to oral penicillin cannot be considered susceptible to cefpodoxime. In addition, parenteral penicillin breakpoints cannot predict cefpodoxime susceptibility but can be used as a complete surrogate marker to infer ceftriaxone susceptibility. Ceftriaxone should not be used as a surrogate for cefpodoxime due to an elevated number of very major errors (false-susceptible). However, ceftriaxone breakpoints lower than the current clinical cutoffs could be specifically applied for categorization of cefpodoxime susceptibility and warrant further evaluation in larger data sets. Similar surrogacy analysis should be conducted for other commonly used oral cephalosporins (e.g., cefdinir and cefuroxime) in the US for treating CABP to provide further guidance to clinical microbiology laboratories.
